# Retraction: Tert-Butylhydroquinone Alleviates Early Brain Injury and Cognitive Dysfunction after Experimental Subarachnoid Hemorrhage: Role of Keap1/Nrf2/ARE Pathway

**DOI:** 10.1371/journal.pone.0355259

**Published:** 2026-08-04

**Authors:** The PLOS One Editors

Following the publication of this article [1], concerns were raised about Figs 2, 8, 10, and the welfare of the rats used in [1]. Specifically:

1. The Fig 2 control panel of this article [1] appears similar to the Fig 1 Control panel in [2] and the Fig 1B Control panel in [3].
2. In Fig 8, the molecular weight reported for the Nrf2 results appears to be incorrect, raising concerns as to whether the results represent the indented protein.
3. The Fig 10A SAH HO-1 panel appears to partially overlap with the Fig 10A SAH+vehicle Keap1 panel. The observed overlap also calls into question the reliability of the associated quantified results presented in Fig 10B.
4. The use of post-operative analgesia following induction of subarachnoid hemorrhage in the rats, predefined humane endpoints, method of euthanasia, and the ethics approval document number were not reported in [1].
5. There are concerns about the use of urethane as an anesthetic in survival surgery due to its toxic effects and about the administration of what appears to be excessive amounts of subcutaneous fluid (57–67 mL/kg) after surgery.

Regarding the first point above, the corresponding author stated that a representative image of a baseline control was unintentionally reused during the figure preparation process. They provided a replacement image for the control panel in Fig 2 from repeat experiments, but PLOS does not consider repeat experiment data sufficient in the absence of original data.

Regarding the second point above, the corresponding author disagreed with the concern, and stated that while it is recognized that Nrf2 can appear at a higher molecular weight (95–110 kDa) due to post-translational modification or electrophoresis conditions, the bands reported in [1] at 68 kDa are consistent with the technical specifications provided by the antibody manufacturer (Abcam, #ab89443) in the conditions described. They indicated that the original blots underlying the published figure are no longer available and instead provided an updated Fig 8 and individual-level data underlying repeat experiments. During editorial assessment of the underlying data provided, unusually repetitive patterns were identified in the dataset, raising concerns about the integrity and reliability of these data.

Regarding the third point above, the corresponding authors stated that the partial overlap in Fig 10 was due to a figure assembly error. They provided a replacement image for Fig 10 from repeat experiments, but PLOS does not consider repeat experiment data sufficient in the absence of original data.

Regarding the fourth point above, the corresponding author provided the ethics approval documents and additional information pertaining to the welfare of the rats used in [1]. Buprenorphine (0.05 mg/kg) was administered subcutaneously every 12 hours for the first 48 hours post-surgery. Animals were monitored every 4 hours for the first 24 hours post-intervention, and then twice daily until the experimental endpoint. Evaluation criteria included respiratory rate, activity level, grooming behavior, posture, and the condition of the surgical incision site. The authors did not provide pre-defined humane endpoints.

Regarding the fifth point above, the corresponding author clarified that use of urethane as an anesthetic was erroneously stated in [1], and that isoflurane was the anesthetic used. They also stated that “twenty” milliliters of 0.9% NaCl was a typographical error and should have read “two” milliliters.

In light of the unresolved concerns regarding Figs 8 and 10, as well as the underlying data, the *PLOS One* Editors retract this article [1].

GC did not agree with the retraction. ZW, CJ, LW, JQ, QL, and ZS either did not respond directly or could not be reached.

This article [1] reports material from [2], which was published in 2013 by Wiley and is not offered under a CC BY license. The Fig 2 Control panel in [1] is not offered under a CC BY license and is therefore excluded from this article’s [1] license.
